# Assessment of Frailty in Hospitalized Cancer Patients: A Psychometric Study of the Brazilian Version of Kihon Checklist

**DOI:** 10.1590/0034-7167-2025-0025

**Published:** 2026-10-19

**Authors:** Paola Alves de Oliveira Lucchesi, Alejandra Rojas-Rivera, Cristiane Helena Gallasch, Eduardo Ferriolli, Renata Eloah de Lucena Ferretti-Rebustini

**Affiliations:** I Universidade de São Paulo. São Paulo, São Paulo, Brazil; II Universidad de los Andes. Santiago, Chile; III Universidade do Estado do Rio de Janeiro. Rio de Janeiro, Rio de Janeiro, Brazil

**Keywords:** Aged, Frailty, Neoplasms, Validation Study, Psychometrics., Anciano, Fragilidad, Neoplasias, Estudio de Validación, Psicometría.

## Abstract

**Objectives:**

to analyze the validity and reliability of Kihon Checklist, Brazilian version, for assessing frailty in older adults hospitalized with cancer.

**Methods:**

psychometric study. Content was analyzed by experts using the Content Validity Ratio. Internal structure was assessed by Exploratory Factor Analysis, Confirmatory Factor Analysis, and reliability. Sample of 250 individuals.

**Results:**

content validity analysis using Content Validity Ratio excluded two items due to values below 0.60. Internal structure analysis showed a better fit in a two-dimensional structure with ten items, named KCL-ONCO10, explaining 66.48% of the phenomenon, with good fit and reliability indices (X^2^=38.98; p=0.04; GFI=0.98; CFI=0.99; AGFI=0.97; RMSEA=0.04; RMSR=0.05; α=0.84; Ω=0.84), but unstable in the construct replicability (GH Index=F1 0.89 - 0.69; F2 0.85 - 0.67).

**Conclusions:**

KCL-ONCO10 demonstrated satisfactory validity and reliability evidence for assessing frailty in older adults hospitalized with cancer.

## RESUMEN

**Objetivos:**

analizar la validez y la fiabilidad de la versión brasileña del *Checklist* Kihon para evaluar la fragilidad en ancianos hospitalizadas por cáncer.

**Métodos:**

estudio psicométrico. Análisis de contenido realizado por expertos mediante el *Content Validity Ratio*. La estructura interna se evaluó mediante Análisis Factorial Exploratorio, Análisis Factorial Confirmatorio y la fiabilidad. Muestra de 250 personas.

**Resultados:**

el análisis de validez de contenido mediante la *Content Validity Ratio* excluyó dos ítems con valores inferiores a 0,60. El análisis de la estructura interna mostró un mejor ajuste en una estructura bidimensional con diez ítems, denominada KCL-ONCO10, explicando el 66,48% del fenómeno, con buenos índices de ajuste y fiabilidad (X^2^=38,98; p=0,04; GFI=0,98; CFI=0,99; AGFI=0,97; RMSEA=0,04; RMSR=0,05; α=0,84; Ω=0,84), pero inestable en la replicabilidad del constructo (índice GH=F1 0,89 - 0,69; F2 0,85 - 0,67).

**Conclusiones:**

el KCL-ONCO10 demostró evidencia satisfactoria de validez y fiabilidad para evaluar la fragilidad en pacientes ancianos hospitalizados con cáncer.

## INTRODUCTION

Cancer is highly prevalent in older adults. Both the development of cancer and the adverse effects of treatment contribute to frailty, which affects about half of older adults with cancer^(1,2)^. In this population, frailty is strongly associated with postoperative complications, increased susceptibility to degree 3 and 4 toxicities, treatment discontinuation, reduced overall survival, hospitalizations, and deaths^(2,3)^.

Frailty is a geriatric syndrome characterized by a state of physiological vulnerability related to aging and a reduction in systemic homeostatic reserve, making individuals more susceptible to stressful events. It is a dynamic phenomenon associated with genetic factors, chronic diseases, history of falls, level of physical activity, and socioeconomic conditions^(4)^.

International societies, such as the International Society of Geriatric Oncology and the American Society of Clinical Oncology, recommend the systematic and continuous assessment of frailty in older adults with cancer in order to personalize treatment and prevent adverse health events^(5,6)^.

Although instruments have been developed and validated for assessing frailty, few present sufficiently robust validity evidence to guarantee their safe use in clinical practice^(7,8)^.

The Kihon Checklist (KCL) is a rapidly administered instrument, developed in Japan, for identifying frailty in older adults. Composed of 25 items, it assesses the lifestyle, physical strength, nutrition, eating, socialization, memory, and mood domains. KCL has been adapted for use in Brazil, Turkey, and Korea^(9,10)^, and its accuracy has been investigated, mainly in outpatient populations, showing a sensitivity of 60% and a specificity of 86.4% for identifying frailty^(6)^.

Despite the cross-cultural adaptation of the Brazilian version of KCL, no studies were identified that assess content validity and internal structure evidence for its use in hospital settings, especially in older adult cancer patients^(7)^.

Validated instruments are essential for the accurate identification and classification of frailty, allowing for more assertive and patient-centered care planning for cancer patients^(4,7)^. Therefore, this study proposes to perform a psychometric analysis of KCL to assess frailty in older adults hospitalized with cancer.

### Study relevance

This article is the product of a dissertation^(11)^ deposited in the institutional repository of the *Universidade de São Paulo* (USP) Digital Library of Theses and Dissertations (https://www.teses.usp.br/teses/disponiveis/7/7139/tde-04112024-175135/pt-br.php).

## OBJECTIVES

To analyze content validity, internal structure, and reliability evidence of the Brazilian version of KCL for assessing frailty in older adults hospitalized with cancer.

## METHODS

### Ethical aspects

The study was conducted in accordance with national and international ethical guidelines and approved by the Research Ethics Committees of the USP School of Nursing and the *Hospital das Clínicas* of the USP School of Medicine, whose opinions accompany this submission. All participants signed the Informed Consent Form.

### Study design, period, and location

This is a psychometric study^(12)^ of the Brazilian version of KCL, carried out between May 2021 and January 2022 and conducted in two phases: the first refers to content validity analysis, carried out in an online format; and the second refers to internal structure and reliability analysis, conducted at a reference institution in the treatment of people with cancer in the state of São Paulo.

### Population or sample; inclusion or exclusion criteria

In the first phase, content validity evidence was investigated through an assessment by a panel of 15 experts, a number estimated based on the critical value of the Content Validity Ratio (CVR) coefficient^(13,14)^. Expert selection was carried out through the *Lattes* platform, considering expertise in geriatrics, gerontology, oncology, and psychometrics.

In the second phase, the modified version of KCL was applied to a sample of 250 participants, following the psychometric recommendation of ten participants per item of the instrument^(15)^. Older adults (age ≥ 60 years) hospitalized in the wards of a cancer referral center, with different types and stages of cancer diagnosis, were included. Individuals hospitalized for COVID-19 and those with terminal illnesses were excluded.

### Study protocol

For content validity evidence analysis, an electronic form available online was sent to experts to assess the items of the Brazilian version of KCL regarding clarity, theoretical relevance, and practical pertinence^(13,16)^, in addition to a characterization questionnaire for these participants, which included variables such as profession, area of activity, and type of current professional activity.

The second phase consisted of applying the modified version of KCL and clinical-demographic characterization instruments to the study’s target population, containing the following variables: sex; age; comorbidities; number of medications in use; oncological diagnosis; reason for hospitalization; last performance result measured by the Karnofsky Performance Scale (KPS)^(17)^, an assessment scale that measures patients’ ability to perform daily tasks without help, estimating tolerance to cancer treatment; and classification regarding the degree of dependence by the Katz Index^(18)^, an instrument used in older adult assessment to determine the level of dependence in performing activities of daily living.

### Analysis of results and statistics

To analyze content validity evidence, the critical CVR estimated at 0.60 (p=0.18) was used, calculated according to the number of experts^(13,14)^. Data were tabulated, and the results for each indicator (clarity, theoretical relevance, and practical pertinence) were analyzed item by item. Items with a CVR value greater than 0.60 were retained. Those with a critical CVR value below the stipulated level were excluded, and items with psychometric inconsistencies descriptively recorded in open field by experts were modified.

Internal structure validity evidence was analyzed using Exploratory Factor Analysis (EFA) and Confirmatory Factor Analysis (CFA)^(19)^. In EFA, data related to responses to KCL items were analyzed using a tetrachoric correlation matrix^(20)^. The instrument’s dimensionality was determined through parallel analysis^(21)^. Factors were extracted using the unweighted robust least squares method with Promin^(22)^ rotation, using a bootstrap of 2,000 cases.

Kaiser Meyer-Olkin Index (KMO) values > 0.50 and Bartlett’s test of sphericity (BTS) p < 0.05^(15)^ were considered adequate to analyze the item set factorability. Unidimensionality was confirmed by Unidimensional Congruence (UniCO) ≥0.95^(15)^. The Explained Common Variance was considered satisfactory ≥ 0.85^(15)^, and the Mean Item Residual Absolute Loadings (MIREAL) were ≤ 0.30^(15)^.

The instrument’s total variance was estimated at the 60% level. Factor loadings with values equal to or greater than 0.35 (for a sample of 250 cases) were considered adequate^(15)^, as well as communalities with values equal to or greater than 0.50^(15)^. The removal of items from the model depended on the magnitude of the factor loadings, communalities and the occurrence of cross-loading (item saturating in more than one factor)^(15)^.

The CFA was performed using the adjusted least squares method for mean and variance. The model fit was analyzed by inspecting the goodness-of-fit indices^(15)^. The assessed fit indices were: non-significant chi-square (x^2^ > 0.05) adjusted for degrees of freedom; Global Fit Index (GFI) ≥ 0.95; Root Mean Square Error of Approximation (RMSEA) ≤ 0.08; standardized Root Mean Square Residual (RMSR) < 0.08; Normalized Fit Index (NFI) ≥ 0.95; Comparative Fit Index (CFI) ≥ 0.97; Adjusted Global Fit Index (AGFI) ≥ 0.95; and Weighted Mean Squared Residual (WRMR) < 1.0^(15)^.

Cronbach’s alpha coefficient and McDonald’s omega coefficient were used as reliability indicators. Values of α > 0.70 and Ω > 0.80 were considered satisfactory^(15)^. To ensure the construct’s replicability, we used the Generalized GH Index (GH > 0.80).

Characterization data (experts and participants) were analyzed using simple descriptive statistics. All analyses were performed using R (version 4.1.3) and Factor (version 12.01.02) software.

## RESULTS

### Phase 1 - Content validity

A panel of experts was composed of 15 professionals, including nurses (47%), physicians (40%), and physiotherapists (13%). Moreover, 33% worked in geriatrics, 20% in oncology and psychometrics, and 13% in gerontology. The majority of them (47%) were involved in teaching or research activities.

Based on experts’ analysis of the 25 items in the instrument, CVR values were calculated and are presented in Table 1. Item 12 was recommended for exclusion due to a lack of clarity, evidenced by the negative CVR (-0.60), and low practical relevance, as indicated by most experts in open field comments. According to them, the item raises doubts by presenting three questions about objective measures (weight, height, and Body Mass Index) without clearly specifying how the answers should be provided. Item 15 was also recommended for exclusion, as it is considered theoretically irrelevant and not relevant in practice for assessing frailty in older adults.

**Table 1:** Kihon Checklist content validity analysis, São Paulo, São Paulo, Brazil

Kihon Checklist Items - Brazilian Version	CVR		
	Cl	TR	PR
***Item 1.*** *Você consegue usar ônibus ou trem sem necessidade de ajuda?*	0.73	1.00	1.00
***Item 2.*** *Você faz compras para o seu dia a dia sem necessidade de ajuda?*	0.87	1.00	1.00
***Item 3.*** *Você administra sua conta/poupança bancária sozinho (a)?*	0.87	1.00	1.00
***Item 4.*** *Você visita à casa de seus amigos?*	0.73	1.00	0.60
***Item 5.*** *Você conversa com seus familiares ou amigos?*	0.87	0.73	0.73
***Item 6.*** *Você sobe escada sem o apoio de corrimão ou parede?*	0.87	1.00	1.00
***Item 7.*** *Você se levanta da cadeira sem usar o braço da mesma como apoio?*	0.73	1.00	1.00
***Item 8.*** *Você caminha mais do que 15 minutos?*	0.07	1.00	1.00
***Item 9.*** *Você sofreu alguma queda (caiu) no último ano?*	1.00	1.00	1.00
***Item 10.*** *Você sente medo de cair?*	1.00	0.87	0.87
***Item 11.*** *Nos últimos 6 meses, você emagreceu 2 a 3 quilos (sem estar de dieta)?*	0.87	1.00	1.00
***Item 12.*** *Qual a sua altura? Qual o seu peso? IMC menor que 18.5?*	- 0.60	0.73	0.73
***Item 13.*** *É correto afirmar que “você não consegue comer alimentos de consistência dura tão bem como 6 meses atrás”?*	0.60	0.87	0.87
***Item 14.*** *Você se engasga quando toma chá ou sopa?*	0.73	1.00	1.00
***Item 15.*** *Você se sente desconfortável com a sensação de boca seca?*	0.73	0.20	0.33
***Item 16.*** *Você sai de casa mais do que uma vez por semana?*	0.87	0.87	0.73
***Item 17.*** *Em comparação ao último ano, você tem saído menos de casa?*	0.73	0.87	0.60
***Item 18.*** *As pessoas têm chamado sua atenção quanto ao seu esquecimento, como: “você faz as mesmas perguntas o tempo todo”?*	0.73	1.00	1.00
***Item 19.*** *Você faz ligações telefônicas checando você mesmo o número de telefone?*	0.07	0.60	0.73
***Item 20.*** *É correto afirmar que “às vezes, você não sabe que dia ou mês é hoje”?*	0.60	0.73	0.73
***Item 21.*** *Nas últimas 2 semanas, você está insatisfeito com sua vida diária?*	1.00	0.60	0.73
***Item 22.*** *Nas últimas 2 semanas, você acha sem graça as atividades com as quais você se divertia antes?*	1.00	0.60	0.87
***Item 23.*** *Nas últimas 2 semanas, você sente dificuldade ao fazer coisas que antes achava fácil de fazer?*	1.00	0.73	0.87
***Item 24.*** *Nas últimas 2 semanas, você sente que não é mais útil para os outros?*	0.87	0.60	0.73
***Item 25.*** *Nas últimas 2 semanas, você se sente exausto sem razão?*	0.87	0.87	1.00

*CVR - Content Validity Ratio; Cl - clarity; TR - theoretical relevance; PR - practical relevance.*

After analyzing the CVR results by indicator and the suggestions from experts recorded in the open field, five items (1, 2, 3, 6, and 9) were retained without changes. Two items (12 and 15) were excluded due to low CVR values in more than one indicator, in addition to observations recorded in the open field. The remaining 18 items were revised to meet evaluators’ recommendations and correct psychometric inconsistencies, with adjustments to verb tense, double question removal, and negative construction removal. Descriptive data from the content validity analysis process are available in the supplementary material (Table 1B - Summary of the process of retaining, modifying, and excluding KCL items after assessment by an expert panel). The pre-final version of KCL consisted of 23 items, proceeding to the second phase of the study.

### Phase 2 - Internal structure and reliability validity

Two hundred and fifty individuals were interviewed, comprising 126 men (50.4%) and 124 women (49.6%). The mean age was 71.20 (±7.41) years. On average, 2.08 (±1.60) comorbidities were identified per person (median = 2.0; IQR: 1.0 - 3.0) and an average of 4.07 (±3.01) types of medications were used (median = 4.0; IQR: 2.0 - 6.0). Most older adults presented partial dependence in performing activities of daily living (n = 104; 41.6%). The majority of participants presented KPS 90 (n = 89; 35.6%).

The most common cancer diagnosis was colon and rectum (n = 51; 20.40%), followed by prostate cancer (n = 30; 12%) and breast cancer (n = 21; 8.40%). The most frequent reasons for hospitalization were curative oncological surgery (n = 82; 33%), adverse events caused by treatment (n = 24; 10%), and oncological emergencies (n = 18; 7%).

Analysis of the internal structure of the modified 23-item KCL version in the first round of EFA confirmed the factorability of the item set (KMO = 0.82 and BTS 253 = 2768.9; p = 0.000010). Parallel analysis indicated that the instrument has only one dimension (UniCo = 0.95; ECV = 0.83; MIREAL = 0.19), with a total explained variance (TEV) of 31.39%.

In terms of factor structure, four items had factor loadings < 0.35, and 20 items had communalities < 0.50. The items most relevant to children of adults were items 9 and 11, with the lowest factor loadings and communalities. Model 1 showed unsatisfactory results in CFA (GFI = 0.877; RMSEA = 0.061; RMSR = 0.103; AGFI = 0.86), but with sufficient reliability levels (α = 0.88; Ω = 0.89) and adequate construct replicability (latent GH = 0.913; observed GH = 0.826).

Due to the inadequacies of the second model, it was decided to explore three dimensions. The third model presented an adequate factorial solution (KMO = 0.819; BTS253 = 2768.9; p = 0.000010).

The VTE of this model was 31.28%, and the factor structure pointed to three items (9, 11, and 12) with low factor loadings in three factors, and again double saturation in item 15. The fit indices showed unsatisfactory results (GFI = 0.943; RMSR = 0.871; AGFI = 0.931), but the model indicated good results for reliability (α = 0.88; Ω = 0.89) and replicability (factor 1: latent GH = 0.830; observed GH = 0.672; factor 2: latent GH = 0.822; observed GH = 0.678; factor 3: latent GH = 0.923; observed GH = 0.800). Table 2 presents a comparative analysis of the three initial models.

**Table 2:** Comparative analysis of models 1, 2, and 3 after factor analysis, São Paulo, São Paulo, Brazil

Item	Model 1		Model 2			Model 3			
	Factor loading	Community	Factor loading		Community	Factor loading			Community
			Factor 1	Factor 2		Factor 1	Factor 2	Factor 3	
1	0.73	0.53	-0.13	0.91	0.66	0.04	-0.18	0.90	0.72
**2**	**0.76**	0.57	-0.21	**1.01**	0.76	-0.07	-0.07	**0.94**	0.76
**3**	**0.61**	0.38	-0.13	**0.77**	0.47	-0.18	0.19	**0.68**	0.51
**4**	**0.48**	0.23	-0.20	**0.69**	0.32	-0.22	0.14	**0.60**	0.35
**5**	**0.39**	0.15	-0.09	**0.49**	0.19	-0.14	0.16	**0.42**	0.21
**6**	**0.43**	0.18	0.19	0.26	0.18	0.26	-0.07	**0.30**	0.21
**7**	**0.61**	0.37	0.15	**0.49**	0.37	0.19	-0.01	**0.50**	0.38
**8**	**0.66**	0.44	-0.03	**0.73**	0.50	0.13	-0.20	**0.75**	0.57
**9**	0.25	0.06	0.17	0.10	0.06	0.06	0.19	0.07	0.07
**10**	**0.43**	0.18	**0.39**	0.08	0.20	0.18	**0.34**	0.06	0.23
**11**	0.19	0.04	-0.03	0.24	0.04	0.09	-0.20	0.28	0.09
**12**	**0.52**	0.27	0.25	**0.31**	0.27	0.14	0.22	0.28	0.27
**13**	**0.42**	0.17	0.24	0.20	0.17	0.01	**0.44**	0.12	0.27
**14**	**0.70**	0.50	**0.30**	**0.45**	0.49	0.29	0.06	**0.47**	0.50
**15**	**0.59**	0.34	**0.86**	-0.17	0.57	**0.59**	**0.38**	-0.10	0.55
**16**	**0.47**	0.22	0.29	0.22	0.23	-0.12	**0.81**	0.07	0.66
**17**	**0.61**	0.37	0.15	**0.49**	0.37	0.03	0.27	**0.43**	0.40
**18**	0.34	0.11	**0.48**	-0.09	0.18	0.13	**0.69**	-0.21	0.43
**19**	0.31	0.09	**0.58**	-0.21	0.21	**0.57**	0.00	-0.12	0.27
**20**	**0.57**	0.32	**0.71**	-0.05	0.45	**0.55**	0.22	0.01	0.45
**21**	**0.39**	0.15	**0.41**	0.03	0.19	**0.52**	-0.16	0.12	0.31
**22**	**0.57**	0.33	**0.74**	-0.07	0.47	**0.71**	0.06	0.01	0.54
**23**	**0.54**	0.29	**0.57**	0.03	0.36	**0.52**	0.09	0.09	0.38

*Note: all factor loadings were presented. Loadings ≥ 0.35 are highlighted in bold.*

Based on the comparative analysis of the presented models, the three-dimensional model indicated a more refined and adjusted internal structure. Therefore, we opted to proceed with factor analysis, maintaining the specifications of the correlation matrix for three factors.

A fourth model revealed a factor solution with a KMO of 0.574 (BTS171 = 2784.2; p = 0.000010), and parallel analysis showed two dimensions. The VTE was 47.84%. Factor structure analysis indicated double saturation of item 6 and low factor loadings for items 5, 7, 8, 10, 13, 14, 16, and 18. CFA of the 19-item model produced satisfactory results for model fit indices and good reliability (α = 0.89; Ω = 0.89), but unstable replicability indicators (factor 1: latent GH = 0.920; observed GH = 0.780; factor 2: latent GH = 0.881; observed GH = 0.704; factor 3: latent GH = 0.859; observed GH = 0.657). This model exhibited many items with low factor loadings and low communalities, leading to the proposal of a new model and excluding the items most characteristic of older adults.

The fifth model tested contained ten items. The sample showed good factorability (KMO=0.67944; BTS45=1212.1; p=0.000010) and two dimensions. The VTE was 66.48%, showing a significant improvement. The demonstrated factor structure was adequate, maintaining high factor loadings for all items, satisfactory results for the model fit indices (X^2^ = 38.98 (p=0.04); GFI=0.987; RMSEA=0.041; RMSR=0.05; NFI=0.989; CFI=0.993; AGFI=0.977; WRMR=0.031) and good reliability (α=0.84; Ω=0.84). However, replicability indicators were unstable (factor 1: latent GH=0.892; observed GH=0.696; factor 2: latent GH=0.851; observed GH=0.673).

After a comparative analysis of the psychometric results for the five models, it was decided to cease the elimination of items and present the ten-item KCL version as the most appropriate. This decision was based on the high factor loadings of items, few items with low common values, better fit through goodness-of-fit indices, and good reliability indices. Furthermore, the two-dimensional model with ten items better preserved its content structure, maintaining conceptual coherence with the construct of frailty in the oncological context. Table 3 presents the factor analysis of models 4 and 5.

**Table 3:** Comparative analysis of models 4 and 5 after exploratory factor analysis, São Paulo, São Paulo, Brazil

Item	Model 4				Model 5		
	Factor loading			Community	Factor loading		Community
	Factor 1	Factor 2	Factor 3		Factor 1	Factor 2	
1	0.84	0.07	-0.13	0.70	0.75	0.15	0.70
**2**	**0.94**	-0.06	-0.08	0.76	**0.88**	0.01	0.79
**3**	**0.68**	-0.17	0.22	0.53	**0.75**	-0.06	0.52
**4**	**0.60**	-0.22	0.16	0.36	**0.58**	-0.09	0.29
**5**	**0.43**	-0.13	0.16	0.21	-	-	-
**6**	**0.39**	**0.51**	-0.12	0.58	-	-	-
**7**	**0.49**	0.23	-0.03	0.41	-	-	-
**8**	**0.67**	0.21	-0.13	0.59	-	-	-
**9**	-	-	-	-	-	-	-
**10**	0.08	0.24	0.29	0.23	-	-	-
**11**	-	-	-	-	-	-	-
**12**	-	-	-	-	-	-	-
**13**	0.11	0.09	**0.39**	0.24	-	-	-
**14**	**0.52**	0.20	0.03	0.46	-	-	-
**15**	-	-	-	-	-	-	-
**16**	0.08	-0.13	**0.88**	0.81	-	-	-
**17**	**0.45**	0.02	0.25	0.38	**0.51**	0.17	0.38
**18**	-0.18	0.19	**0.64**	0.40	-	-	-
**19**	-0.16	**0.54**	0.07	0.24	-0.11	**0.64**	0.34
**20**	0.02	**0.49**	0.24	0.38	0.07	**0.57**	0.38
**21**	0.11	**0.84**	-0.18	0.77	-0.01	**0.81**	0.65
**22**	-0.01	**0.69**	0.11	0.52	-0.08	**0.80**	0.59
**23**	0.02	**0.62**	0.12	0.45	0.06	**0.57**	0.37

*Note: all factor loadings have been presented. Loadings ≥ 0.35 are highlighted in bold; “ -” indicates that the item was not included in the factor analysis of the presented model.*

Parallel analysis of the final model indicated two dimensions. The first was termed “instrumental activities”, comprising items 1, 2, 3, 4, and 17, and the second, “depressed mood”, comprising items 19, 20, 21, 22, and 23.

Thus, after analyzing internal structure evidence, a new version of KCL emerged for measuring frailty in cancer patients with two dimensions, composed of ten items (the first five measure the “instrumental activities” dimension, and the last five, the “mood” dimension), considered the best version, which was named Kihon Checklist-ONCO10 (KCL-ONCO10), presented in Chart 1.

**Chart 1:** Ten-item version of the Kihon Checklist (KCL-ONCO10) for measuring frailty in hospitalized cancer patients, São Paulo, São Paulo, Brazil

Kihon Checklist (KCL-ONCO	
**1**	*Você consegue usar ônibus, trem ou outro meio de transporte sem necessidade de ajuda?*
**2**	*Você faz compras para o seu dia a dia sem necessidade de ajuda?*
**3**	*Você administra sua conta bancária sozinho (a)?*
**4**	*Você visita a casa de seus amigos?*
**5**	*Você faz ligações telefônicas sozinho, checando você mesmo o número de telefone?*
**6**	*Nas últimas 2 semanas, você tem se sentido insatisfeito com sua vida diária?*
**7**	*Nas últimas 2 semanas, você tem achado sem graça as atividades com as quais você se divertia antes?*
**8**	*Nas últimas 2 semanas, você tem sentido dificuldade ao fazer coisas que antes achava fácil de fazer?*
**9**	*Nas últimas 2 semanas, você tem se sentido sem utilidade para os outros?*
**10**	*Nas últimas 2 semanas, você tem se sentido cansado sem motivo?*

## DISCUSSION

In oncology, disease progression and treatment-related toxicities challenge organic homeostatic processes. In older adults, these processes can be even more costly when we consider their increased vulnerability to disease due to the processes of aging itself^(23)^.

In daily clinical practice, scales that assess patients’ performance status, such as the KPS and the Eastern Cooperative Oncology Group, are commonly used. However, these scales have limitations when applied to older adults. They underrepresent the functional status of these patients and have lower inter-observer agreement, while frailty assessment instruments can assess a person’s long-term resilience and reserve^(24)^.

The presence of frailty in older adults with cancer is associated with numerous negative health outcomes such as falls, long-term functional decline, higher rates of surgical complications and hospital readmission when identified at hospital discharge after cancer surgery^(25)^, in addition to being related to decreased quality of life and mental health problems in older adult cancer survivors^(26,27)^.

In this context, frailty becomes an indicator of treatment tolerance and a guideline for an individualized care plan^(4)^. Therefore, this study advocates the use of a frailty assessment instrument with metric properties tested in an oncology context to ensure more reliable and trustworthy results in clinical practice.

The KCL content validity was established through CVR analysis, a robust indicator derived from expert assessments of the adequacy of items in terms of clarity, theoretical relevance, and practical pertinence. According to Lawshe, this indicator confidently guarantees content validity^(13,14)^.

Although demonstrating an adequate factor solution, it revealed dimensions different from those reported by the authors of the original instrument, encountering several structural problems. The initial model identified numerous items with low factor loadings and communalities. Some hypotheses for the low scores on indicators may be attributed to factors intrinsic to oncological disease, which may be common and reduce the variance of the responses. This highlights the importance of revisiting such evidence, especially regarding the internal structure^(28)^.

The findings revealed that the optimal structure for KCL comprises ten items, distributed across two dimensions (instrumental and psychological), assessing 66.48% of frailty in older adult cancer patients, with strong validity and reliability evidence. For this version to be properly used, testing for associations with other variables and test consequences is recommended, completing the arc of validity evidence^(4,28)^.

### Study limitations

Although we found a favorable factor solution for KCL-ONCO10, the instability in the construct replicability represents a limitation. Therefore, replication of the study in larger and more diverse samples is recommended to confirm the model’s stability. Despite this, KCL-ONCO10 demonstrates good validity and reliability evidence for assessing older adults with similar profiles.

### Contributions to nursing, health, or public policy

This study offers an instrument with strong validity evidence, which contributes to the assessment of frailty in older adults hospitalized with cancer, supporting care planning by the nursing team and strengthening individualized clinical decisions. Furthermore, the study advances good practices in instrument validity by using contemporary psychometric techniques that reinforce the robustness of results.

## CONCLUSIONS

The Brazilian version of KCL with 25 items does not present good content validity and internal structure evidence in older adult cancer patients. However, KCL-ONCO10, comprising ten items in two dimensions (instrumental activities and mood), demonstrates good content validity, internal structure, and reliability evidence for assessing frailty in hospitalized older adult cancer patients.

## Data Availability

The research data are available in a repository: https://doi.org/10.48331/SCIELODATA.YYI2XL.

## References

[1] Komici K, Bencivenga L, Navani N, D’Agnano V, Guerra G, Bianco A (2022). Frailty in patients with lung cancer: a systematic review and meta-analysis. Chest.

[2] Wang S, Yang T, Qiang W, Shen A, Zhao Z, Yang H (2022). The prevalence of frailty among breast cancer patients: a systematic review and meta-analysis. Support Care Cancer.

[3] Tang WZ, Tan ZK, Qiu LY, Chen JQ, Jia K. (2024). Prevalence and unfavorable outcome of frailty in older adults with gastric cancer: a systematic review and meta-analysis. Support Care Cancer.

[4] Ferrioli E, Fernandes PMP. (2024). Frailty syndrome and healthcare for older adults. Sao Paulo Med J.

[5] Dale W, Klepin HD, Williams GR, Alibhai SMH, Bergerot C, Brintzenhofeszoc K (2023). Practical assessment and management of vulnerabilities in older patients receiving systemic cancer therapy: ASCO guideline update. J Clin Oncol.

[6] Goede V. (2023). Frailty and cancer: current perspectives on assessment and monitoring. Clin Interv Aging.

[7] Ferretti-Rebustini REL, Lucchesi PAO, Alves WS, Rojas-Rivera A, Silva DF, Ribeiro JB (2024). Instrumentos de avaliação multidimensional de fragilidade para uso no cuidado de idosos com doença cardiovascular. Rev Enferm UERJ.

[8] Sutton JL, Gould RL, Daley S, Coulson MC, Ward EV, Butler AM (2016). Psychometric properties of multicomponent tools designed to assess frailty in older adults: a systematic review. BMC Geriatr.

[9] Satake S, Senda K, Hong YJ, Miura H, Endo H, Sakurai T (2016). Validity of the Kihon Checklist for assessing frailty status. Geriatr Gerontol Int.

[10] Sewo Sampaio PY, Sampaio RA, Yamada M, Ogita M, Arai H. (2014). Validation and translation of the Kihon Checklist (frailty index) into Brazilian Portuguese. Geriatr Gerontol Int.

[11] Lucchesi PAO. (2022). Validação do Kihon: checklist para mensuração da fragilidade em idosos com câncer hospitalizados.

[12] Furr RM., Furr RM. (2018). Psychometrics: an introduction.

[13] Ferretti-Rebustini REL. (2023). Psychometrics: applications in nursing. Rev Latino-Am Enfermagem.

[14] Almanasreh E, Moles R, Chen TF. (2019). Evaluation of methods used for estimating content validity. Res Social Adm Pharm.

[15] Ayre C, Scally AJ. (2014). Critical values for lawshe’s content validity ratio: revisiting the original methods of calculation. Meas Eval Couns Dez.

[16] Alexandre NMC, Coluci MZO. (2011). Validade de conteúdo nos processos de construção e adaptação de instrumentos de medidas. Ciênc Saúde Coletiva.

[17] Hair JF, Black WC, Babin BJ, Anderson RE., Hair JF, Black WC, Babin BJ, Anderson RE. (2018). Multivariate data analysis.

[18] Ferreira PCDS, Moreira MN, Lourenço RA. (2024). Cross-cultural adaptation of the Karnofsky Performance Status instrument to Brazilian Portuguese. Rev Col Bras Cir.

[19] Gallasch CH, Vieira HWD, Lucchesi PAO, Balbinotti MAA, Rebustini F, Ferretti-Rebustini REL. (2022). Validade do Índice de Katz para avaliar a dependência em pacientes em tratamento oncológico. Acta Paul Enferm.

[20] Gaskin CJ, Happell B. (2014). On exploratory factor analysis: a review of recent evidence, an assessment of current practice, and recommendations for future use. Int J Nurs Stud.

[21] Finch WH. (2023). A comparison of methods for determining the number of factors to retain with exploratory factor analysis of dichotomous data. Psych.

[22] Timmerman ME, Lorenzo-Seva U. (2011). Dimensionality assessment of ordered polytomous items with parallel analysis. Psychol Methods.

[23] Watkins MW. (2018). Exploratory factor analysis: a guide for best practice. J Black Psychol.

[24] Pergolotti M, Wood KC, Hidde M, Kendig TD, Ronnen EA, Giri S (2024). Geriatric assessment-identified impairments and frailty in adults with cancer younger than 65: an opportunity to optimize oncology care. J Geriatr Oncol.

[25] Guven DC, Martinez-Cannon BA, Testa GD, Martins JC, Velasco RN Jr, Kalsi T (2024). Immunotherapy use in older adults with cancer with frailty: a young SIOG review paper. J Geriatr Oncol.

[26] Miao X, Guo Y, Chen Y, Xu X, Ding L, Hu J (2024). Exploring frailty trajectories and their associations with health outcomes in older people. Eur J Surg Oncol.

[27] Hassan AM, Paidisetty P, Ray N, Govande JG, Nelson JA, Mehrara BJ (2024). Frailty in autologous breast reconstruction: impact on outcomes. Ann Surg Oncol.

[28] Meissner VH, Imhof K, Jahnen M, Lunger L, Dinkel A, Schiele S (2023). Prevalence of frailty in long-term prostate cancer survivors after radical prostatectomy and its association with quality of life and emotional health. Ann Oncol.

