# Supplementary material for: Assessment of Frailty in Hospitalized Cancer Patients: A Psychometric Study of the Brazilian Version of Kihon Checklist
Source: Rev Bras Enferm. 2026 Oct 19;79:e20250025. doi: 10.1590/0034-7167-2025-0025 (PMC13679588; doi:10.1590/0034-7167-2025-0025)
Supplement: Translation

# Translation: Avaliação da Fragilidade em Pacientes Oncológicos Hospitalizados: Estudo Psicométrico da Versão Brasileira do *Kihon Checklist*

**DOI:** 10.1590/0034-7167-2025-0025pt

**Authors:** Paola Alves de Oliveira Lucchesi, Alejandra Rojas-Rivera, Cristiane Helena Gallasch, Eduardo Ferriolli, Renata Eloah de Lucena Ferretti-Rebustini

**Affiliations:** I Universidade de São Paulo. São Paulo, São Paulo, Brasil; II Universidad de los Andes. Santiago, Chile; III Universidade do Estado do Rio de Janeiro. Rio de Janeiro, Rio de Janeiro, Brasil

**Keywords:** Idoso, Fragilidade, Neoplasias, Estudo de Validação, Psicometria.

## Abstract

**Objetivos:**

analisar a validade e confiabilidade da versão brasileira do Kihon *Checklist* para avaliação da fragilidade em pessoas idosas hospitalizadas com câncer.

**Métodos:**

estudo psicométrico. Conteúdo analisado por especialistas pelo *Content Validity Ratio*. Estrutura interna avaliada por Análise Fatorial Exploratória, Confirmatória e confiabilidade. Amostra de 250 indivíduos.

**Resultados:**

análise da validade de conteúdo por *Content Validity Ratio* excluiu dois itens por valor abaixo de 0,60. Análise da estrutura interna observou melhor ajuste em estrutura bidimensional com dez itens, denominado KCL-ONCO10, explicando 66,48% do fenômeno, bons índices de ajuste e confiabilidade (X^2^=38,98; p=0,04; GFI=0,98; CFI=0,99; AGFI=0,97; RMSEA=0,04; RMSR=0,05; α=0,84; Ω=0,84), mas instável na replicabilidade do construto (Índice GH=F1 0,89 - 0,69; F2 0,85 - 0,67).

**Conclusões:**

o KCL-ONCO10 demonstrou evidências satisfatórias de validade e confiabilidade para avaliar fragilidade em idosos hospitalizados com câncer.

## INTRODUÇÃO

O câncer é altamente prevalente na população idosa. Tanto o desenvolvimento da doença oncológica quanto os efeitos adversos decorrentes do tratamento contribuem para o surgimento da fragilidade, que acomete cerca de metade das pessoas idosas com câncer^(1,2)^. Nessa população, a fragilidade está fortemente associada a complicações pós-operatórias, maior suscetibilidade a toxicidades de grau 3 e 4, descontinuação do tratamento, redução da sobrevida global, hospitalizações, e óbitos^(2,3)^.

A fragilidade é uma síndrome geriátrica caracterizada por um estado de vulnerabilidade fisiológica relacionado ao envelhecimento e à redução da reserva homeostática sistêmica, tornando os indivíduos mais suscetíveis a eventos estressores. Trata-se de fenômeno dinâmico, associado a fatores genéticos, doenças crônicas, histórico de quedas, nível de atividade física e condições socioeconômicas^(4)^.

Sociedades internacionais, como a Sociedade Internacional de Oncologia Geriátrica e a Sociedade Americana de Oncologia Clínica, recomendam a avaliação sistemática e contínua da fragilidade em pessoas idosas com câncer com o objetivo de personalizar o tratamento e prevenir a ocorrência de eventos adversos à saúde^(5,6)^.

Embora existam instrumentos desenvolvidos e validados para a avaliação da fragilidade, poucos apresentam evidências de validade suficientemente robustas para garantir seu uso seguro na prática clínica^(7,8)^.

O *Kihon Checklist* (KCL) é um instrumento de rápida aplicação, desenvolvido no Japão, para a identificação da fragilidade em pessoas idosas. Composto por 25 itens, avalia os domínios de estilo de vida, força física, nutrição, dieta, socialização, memória, cognição e humor depressivo. O KCL foi adaptado para uso no Brasil, Turquia e Coreia^(9,10)^, e sua acurácia tem sido investigada, principalmente, em populações ambulatoriais, apresentando sensibilidade de 60% e especificidade de 86,4% para a identificação da fragilidade^(6)^.

Apesar da adaptação transcultural da versão brasileira do KCL, não foram identificados estudos que avaliem as evidências de validade de conteúdo e de estrutura interna para seu uso em contextos hospitalares, especialmente em pacientes idosos com câncer^(7)^.

Instrumentos validados são essenciais para a identificação e classificação precisa da fragilidade, permitindo um planejamento de cuidado mais assertivo e centrado no paciente oncológico^(4,7)^. Diante disso, este estudo propõe realizar a análise psicométrica do KCL para avaliação da fragilidade em pessoas idosas hospitalizadas com câncer.

### Relevância do estudo

Este artigo é produto de dissertação^(11)^ depositada no repositório institucional da Biblioteca Digital de Teses e Dissertações da Universidade de São Paulo (USP) (https://www.teses.usp.br/teses/disponiveis/7/7139/tde-04112024-175135/pt-br.php).

## OBJETIVOS

Analisar as evidências de validade de conteúdo, estrutura interna e confiabilidade da versão brasileira do KCL para a avaliação da fragilidade em pessoas idosas hospitalizadas com câncer.

## MÉTODOS

### Aspectos éticos

O estudo foi conduzido em conformidade com as diretrizes éticas nacionais e internacionais, e aprovado pelos Comitês de Ética em Pesquisa da Escola de Enfermagem da USP e do Hospital das Clínicas da Faculdade de Medicina da USP, cujos pareceres acompanham esta submissão. Todos os participantes assinaram o Termo de Consentimento Livre e Esclarecido.

### Desenho, período e local do estudo

Trata-se de estudo psicométrico^(12)^ da versão brasileira do KCL, realizado entre maio de 2021 e janeiro de 2022 e conduzido em duas fases: a primeira é referente à análise de validade de conteúdo, realizada em formato *online*; e a segunda é referente à análise da estrutura interna e confiabilidade, conduzida em uma instituição de referência no tratamento de pessoas com câncer do estado de São Paulo.

### População ou amostra; critérios de inclusão ou exclusão

Na primeira fase, as evidências de validade de conteúdo foram investigadas a partir da avaliação de um painel composto por 15 especialistas, número estimado com base no valor crítico do coeficiente *Content Validity Ratio* (CVR)^(13,14)^. A seleção dos especialistas foi realizada por meio da plataforma *Lattes*, considerando *expertise* nas áreas da geriatria, gerontologia, oncologia e psicometria.

Na segunda fase, a versão modificada do KCL foi aplicada a uma amostra de 250 participantes, seguindo a recomendação psicométrica de dez participantes por item do instrumento^(15)^. Foram incluídas pessoas idosas (idade ≥ 60 anos), hospitalizadas nas enfermarias de um centro de referência em câncer, com diferentes tipos e estágios de diagnóstico oncológico. Foram excluídos indivíduos internados por COVID-19 e aqueles em terminalidade de vida.

### Protocolo do estudo

Para a análise das evidências de validade de conteúdo, foi enviado aos especialistas um formulário eletrônico disponível *online* para avaliação dos itens da versão brasileira do KCL quanto à clareza, relevância teórica e pertinência prática^(13,16)^, além de questionário de caracterização desses participantes, que incluíram as variáveis profissão, área de atuação e tipo de atividade profissional atual.

A segunda fase consistiu em aplicar a versão modificada do KCL e instrumentos de caracterização clínico-demográfica, junto à população alvo do estudo, contendo as seguintes variáveis: sexo; idade; comorbidades; número de medicamentos em uso; diagnóstico oncológico; motivo da internação; último resultado de performance medido pela *Karnofsky Performance Scale* (KPS)^(17)^, escala de avaliação que mensura a capacidade do paciente em realizar tarefas cotidianas sem ajuda, estimando a tolerância ao tratamento oncológico; e a classificação quanto ao grau de dependência pelo Índice de Katz^(18)^, instrumento utilizado na avaliação da pessoa idosa para determinar o nível de dependência para realizar atividades básicas de vida diária.

### Análise dos resultados e estatística

Para analisar as evidências de validade de conteúdo, foi utilizado o CVR crítico estimado em 0,60 (p=0,18), calculado de acordo com o número de especialistas^(13,14)^. Os dados foram tabulados, e os resultados de cada indicador (clareza, relevância teórica e pertinência prática), analisados item a item. Foram mantidos os itens com valor de CVR maior que 0,60. Aqueles com valor de CVR crítico abaixo do estipulado foram excluídos, e os itens com inconsistências psicométricas registradas descritivamente em campo aberto pelos especialistas, modificados.

As evidências de validade de estrutura interna foram analisadas por meio da Análise Fatorial Exploratória (AFE) e da Análise Fatorial Confirmatória (AFC)^(19)^. Na AFE, os dados relacionados às respostas aos itens do KCL foram analisados por meio de uma matriz de correlação tetracórica^(20)^. A dimensionalidade do instrumento foi determinada por meio de análise paralela^(21)^. Os fatores foram extraídos pelo método dos mínimos quadrados robustos não ponderados, com rotação Promin^(22)^, sendo utilizado um *bootstrap* de 2.000 casos.

Valores do Índice de Kaiser Meyer-Olkin (KMO) > 0,50 e do teste de esfericidade de Bartlett (TEB) p < 0,05^(15)^ foram considerados adequados para analisar a fatorabilidade do conjunto de itens. A confirmação da unidimensionalidade foi verificada pela *Unidimensional Congruence* (UniCO) ≥0,95^(15)^. A *Explained Common Variance* (ECV) foi considerada satisfatória ≥ 0,85^(15)^, e as *Mean Item Residual Absolute Loadings* (MIREAL) foram ≤ 0,30^(15)^.

A variância total do instrumento foi estimada ao nível de 60%. Cargas fatoriais com valores iguais ou superiores a 0,35 (para uma amostra de 250 casos) foram consideradas adequadas^(15)^, além de comunalidades com valores iguais ou superiores a 0,50^(15)^. A retirada de itens do modelo dependeu da magnitude das cargas fatoriais, comunalidades e da ocorrência de *cross loading* (item saturando em mais de um fator)^(15)^.

A AFC foi realizada pelo método dos mínimos quadrados ajustados para média e variância. O ajuste do modelo foi analisado pela inspeção dos índices de qualidade do ajuste^(15)^. Os índices de ajuste avaliados foram: qui-quadrado não significativo (x^2^ > 0,05) ajustado por graus de liberdade; *Global Fit Index* (GFI) ≥ 0,95; *Root Mean Square Error of Approximation* (RMSEA) ≤ 0,08; *standardized Root Mean Square Residual* (RMSR) < 0,08; *Normalized Fit Index* (NFI) ≥ 0,95; *Comparative Fit Index* (CFI) ≥ 0,97; *Adjusted Global Fit Index* (AGFI) ≥ 0,95; e *Weighted Mean Squared Residual* (WRMR) < 1,0^(15)^.

O coeficiente alfa de Cronbach e o ômega de McDonald foram utilizados como indicadores de confiabilidade. Valores de α > 0,70 e Ω > 0,80 foram considerados satisfatórios^(15)^. Para garantir a replicabilidade do construto, utilizamos o Índice GH Generalizado (GH > 0,80).

Os dados de caracterização (especialistas e participantes) foram analisados por meio de estatística descritiva simples. Todas as análises foram feitas usando os *softwares* R (versão 4.1.3) e *Factor* (versão 12.01.02).

## RESULTADOS

### Fase 1 - Validade de conteúdo

O painel de especialistas foi composto por 15 profissionais, entre enfermeiros (47%), médicos (40%) e fisioterapeutas (13%). Atuavam em geriatria 33%, em oncologia e psicometria, 20%, e em gerontologia, 13%. A maior parte deles (47%) estava envolvido em atividades de ensino ou pesquisa.

A partir da análise dos especialistas referente aos 25 itens do instrumento, os valores de CVR foram calculados e estão apresentados na Tabela 1. O item 12 foi recomendado para exclusão devido à falta de clareza, evidenciada pelo CVR negativo (-0,60), e à baixa pertinência prática, apontada pela maioria dos especialistas nos comentários em campo aberto. Segundo eles, o item gera dúvidas por apresentar três perguntas sobre medidas objetivas (peso, altura e Índice de Massa Corporal), sem especificar claramente como as respostas deveriam ser fornecidas. O item 15 também foi recomendado para exclusão, por ser considerado teoricamente irrelevante e não pertinente na prática para avaliar a fragilidade em pessoas idosas.

**Tabela 1:** Análise da Validade de Conteúdo do *Kihon Checklist*, São Paulo, São Paulo, Brasil

Itens do *Kihon Checklist* - Versão Brasileira	CVR		
	Cl	RT	PP
Item 1. Você consegue usar ônibus ou trem sem necessidade de ajuda?	0,73	1,00	1,00
**Item 2.** Você faz compras para o seu dia a dia sem necessidade de ajuda?	0,87	1,00	1,00
**Item 3.** Você administra sua conta/poupança bancária sozinho (a)?	0,87	1,00	1,00
**Item 4.** Você visita à casa de seus amigos?	0,73	1,00	0,60
**Item 5.** Você conversa com seus familiares ou amigos?	0,87	0,73	0,73
**Item 6.** Você sobe escada sem o apoio de corrimão ou parede?	0,87	1,00	1,00
**Item 7.** Você se levanta da cadeira sem usar o braço da mesma como apoio?	0,73	1,00	1,00
**Item 8.** Você caminha mais do que 15 minutos?	0,07	1,00	1,00
**Item 9.** Você sofreu alguma queda (caiu) no último ano?	1,00	1,00	1,00
**Item 10.** Você sente medo de cair?	1,00	0,87	0,87
**Item 11.** Nos últimos 6 meses, você emagreceu 2 a 3 quilos (sem estar de dieta)?	0,87	1,00	1,00
**Item 12.** Qual a sua altura? Qual o seu peso? IMC menor que 18.5?	- 0,60	0,73	0,73
**Item 13.** É correto afirmar que “você não consegue comer alimentos de consistência dura tão bem como 6 meses atrás”?	0,60	0,87	0,87
**Item 14.** Você se engasga quando toma chá ou sopa?	0,73	1,00	1,00
**Item 15.** Você se sente desconfortável com a sensação de boca seca?	0,73	0,20	0,33
**Item 16.** Você sai de casa mais do que uma vez por semana?	0,87	0,87	0,73
**Item 17.** Em comparação ao último ano, você tem saído menos de casa?	0,73	0,87	0,60
**Item 18.** As pessoas têm chamado sua atenção quanto ao seu esquecimento, como: “você faz as mesmas perguntas o tempo todo”?	0,73	1,00	1,00
**Item 19.** Você faz ligações telefônicas checando você mesmo o número de telefone?	0,07	0,60	0,73
**Item 20.** É correto afirmar que “às vezes, você não sabe que dia ou mês é hoje”?	0,60	0,73	0,73
**Item 21.** Nas últimas 2 semanas, você está insatisfeito com sua vida diária?	1,00	0,60	0,73
**Item 22.** Nas últimas 2 semanas, você acha sem graça as atividades com as quais você se divertia antes?	1,00	0,60	0,87
**Item 23.** Nas últimas 2 semanas, você sente dificuldade ao fazer coisas que antes achava fácil de fazer?	1,00	0,73	0,87
**Item 24.** Nas últimas 2 semanas, você sente que não é mais útil para os outros?	0,87	0,60	0,73
**Item 25.** Nas últimas 2 semanas, você se sente exausto sem razão?	0,87	0,87	1,00

*CVR - Content Validity Ratio; Cl - clareza; RT - relevância teórica; PP - pertinência prática.*

Após a análise dos resultados do CVR por indicador e das sugestões dos especialistas registradas em campo aberto, cinco itens (1, 2, 3, 6 e 9) foram mantidos sem alterações. Dois itens (12 e 15) foram excluídos devido aos baixos valores de CVR em mais de um indicador, além das observações registradas em campo aberto. Os demais 18 itens foram revisados para atender às recomendações dos avaliadores e corrigir inconsistências psicométricas, com ajustes no tempo verbal, eliminação de perguntas duplas e remoção de construções negativas. Os dados descritivos do processo de análise da validade de conteúdo estão disponíveis no material suplementar (Tabela 1B - Resumo do processo de retenção, modificação e exclusão dos itens do KCL após avaliação do painel de especialistas). A versão pré-final do KCL foi composta por 23 itens, seguindo para a segunda fase do estudo.

### Fase 2 - Validação da estrutura interna e confiabilidade

Foram entrevistados 250 indivíduos, sendo 126 homens (50,4%) e 124 mulheres (49,6%). A média de idade foi de 71,20 (±7,41) anos. Em média, foram identificados 2,08 (±1,60) comorbidades por pessoa (mediana = 2,0; IIQ: 1,0 - 3,0) e uso médio de 4,07 (±3,01) tipos de medicamentos (mediana = 4,0; IIQ: 2,0 - 6,0). A maioria das pessoas idosas apresentava dependência parcial para desenvolver atividades básicas de vida diária (n = 104; 41,6%). A maior parte dos participantes apresentou KPS 90 (n = 89; 35,6%).

O diagnóstico de câncer mais comum foi de cólon e reto (n = 51; 20,40%), seguido de câncer de próstata (n = 30; 12%) e câncer de mama (n = 21; 8,40%). Os motivos de internação mais frequentes foram cirurgia oncológica curativa (n = 82; 33%), evento adverso provocado pelo tratamento (n = 24; 10%) e emergências oncológicas (n=18; 7%).

A análise da estrutura interna da versão modificada do KCL, com 23 itens, na primeira rodada da AFE, confirmou a fatorabilidade do conjunto de itens (KMO = 0,82 e TEB 253 = 2768,9; p = 0,000010). A análise paralela indicou que o instrumento possui apenas uma dimensão (UniCo = 0,95; ECV = 0,83; MIREAL = 0,19), com uma variância total explicada (VTE) de 31,39%.

Em termos de estrutura fatorial, quatro itens apresentaram cargas fatoriais < 0,35, e 20 itens apresentaram comunalidades < 0,50. Os itens mais ruidosos foram os itens 9 e 11, com as menores cargas fatoriais e comunalidades. O modelo 1 apresentou resultados insatisfatórios na AFC (GFI = 0,877; RMSEA = 0,061; RMSR = 0,103; AGFI = 0,86), mas com níveis de confiabilidade suficientes (α = 0,88; Ω = 0,89) e replicabilidade do construto adequada (GH latente = 0,913; GH observado = 0,826).

Devido às inadequações do segundo modelo, decidiu-se explorar três dimensões. O terceiro modelo apresentou solução fatorial adequada (KMO = 0,819; TEB253 = 2768,9; p = 0,000010).

O VTE deste modelo foi de 31,28%, e a estrutura fatorial apontou para três itens (9, 11 e 12) com cargas fatoriais baixas em três fatores, e novamente saturação dupla no item 15. Os índices de ajuste mostraram resultados insatisfatórios (GFI = 0,943; RMSR = 0,871; AGFI = 0,931), mas o modelo indicou bons resultados para confiabilidade (α = 0,88; Ω = 0,89) e replicabilidade (fator 1: GH latente = 0,830; GH observado = 0,672; fator 2: GH latente = 0,822; GH observado = 0,678; fator 3: GH latente = 0,923; GH observado = 0,800). A Tabela 2 apresenta uma análise comparativa dos três modelos iniciais.

**Tabela 2:** Análise comparativa dos modelos 1, 2 e 3 após análise fatorial, São Paulo, São Paulo, Brasil

Item	Modelo 1		Modelo 2			Modelo 3			
	Carga fatorial	Comunalidade	Carga fatorial		Comunalidade	Carga fatorial			Comunalidade
			Fator 1	Fator 2		Fator 1	Fator 2	Fator 3	
1	0,73	0,53	-0,13	0,91	0,66	0,04	-0,18	0,90	0,72
**2**	**0,76**	0,57	-0,21	**1,01**	0,76	-0,07	-0,07	**0,94**	0,76
**3**	**0,61**	0,38	-0,13	**0,77**	0,47	-0,18	0,19	**0,68**	0,51
**4**	**0,48**	0,23	-0,20	**0,69**	0,32	-0,22	0,14	**0,60**	0,35
**5**	**0,39**	0,15	-0,09	**0,49**	0,19	-0,14	0,16	**0,42**	0,21
**6**	**0,43**	0,18	0,19	0,26	0,18	0,26	-0,07	**0,30**	0,21
**7**	**0,61**	0,37	0,15	**0,49**	0,37	0,19	-0,01	**0,50**	0,38
**8**	**0,66**	0,44	-0,03	**0,73**	0,50	0,13	-0,20	**0,75**	0,57
**9**	0,25	0,06	0,17	0,10	0,06	0,06	0,19	0,07	0,07
**10**	**0,43**	0,18	**0,39**	0,08	0,20	0,18	**0,34**	0,06	0,23
**11**	0,19	0,04	-0,03	0,24	0,04	0,09	-0,20	0,28	0,09
**12**	**0,52**	0,27	0,25	**0,31**	0,27	0,14	0,22	0,28	0,27
**13**	**0,42**	0,17	0,24	0,20	0,17	0,01	**0,44**	0,12	0,27
**14**	**0,70**	0,50	**0,30**	**0,45**	0,49	0,29	0,06	**0,47**	0,50
**15**	**0,59**	0,34	**0,86**	-0,17	0,57	**0,59**	**0,38**	-0,10	0,55
**16**	**0,47**	0,22	0,29	0,22	0,23	-0,12	**0,81**	0,07	0,66
**17**	**0,61**	0,37	0,15	**0,49**	0,37	0,03	0,27	**0,43**	0,40
**18**	0,34	0,11	**0,48**	-0,09	0,18	0,13	**0,69**	-0,21	0,43
**19**	0,31	0,09	**0,58**	-0,21	0,21	**0,57**	0,00	-0,12	0,27
**20**	**0,57**	0,32	**0,71**	-0,05	0,45	**0,55**	0,22	0,01	0,45
**21**	**0,39**	0,15	**0,41**	0,03	0,19	**0,52**	-0,16	0,12	0,31
**22**	**0,57**	0,33	**0,74**	-0,07	0,47	**0,71**	0,06	0,01	0,54
**23**	**0,54**	0,29	**0,57**	0,03	0,36	**0,52**	0,09	0,09	0,38

*Nota: todas as cargas fatoriais foram apresentadas. Estão destacadas em negrito as cargas ≥ 0,35.*

Com base na análise comparativa dos modelos apresentados, o tridimensional indicou uma estrutura interna mais aprimorada e ajustada. Portanto, optamos por prosseguir com a análise fatorial, mantendo as especificações da matriz de correlação para três fatores.

Um quarto modelo revelou uma solução fatorial com um KMO de 0,574 (TEB171= 2784,2; p=0,000010), e a análise paralela mostrou duas dimensões. A VTE foi de 47,84%. A análise da estrutura fatorial indicou saturação dupla do item 6 e baixas cargas fatoriais para os itens 5, 7, 8, 10, 13, 14, 16 e 18. A AFC do modelo com 19 itens produziu resultados satisfatórios para índices de ajuste do modelo e boa confiabilidade (α=0,89; Ω=0,89), mas indicadores de replicabilidade instáveis (fator 1: GH latente=0,920; GH observado=0,780; fator 2: GH latente=0,881; GH observado=0,704; fator 3: GH latente=0,859; GH observado=0,657). Este modelo exibiu muitos itens com baixas cargas fatoriais e baixas comunalidades, levando à proposta de um novo modelo e excluindo os itens mais ruidosos.

O quinto modelo testado continha dez itens. A amostra apresentou boa fatorabilidade (KMO=0,67944; TEB45=1212,1; p=0,000010) e duas dimensões. A VTE foi de 66,48%, apresentando melhora significativa. A estrutura fatorial demonstrada foi adequada, mantendo altas cargas fatoriais para todos os itens, resultados satisfatórios para os índices de ajuste do modelo (X^2^ = 38,98 (p=0,04); GFI=0,987; RMSEA=0,041; RMSR=0,05; NFI=0,989; CFI=0,993; AGFI=0,977; WRMR=0,031) e boa confiabilidade (α=0,84; Ω=0,84). Entretanto, os indicadores de replicabilidade foram instáveis (fator 1: GH latente=0,892; GH observado=0,696; fator 2: GH latente=0,851; GH observado=0,673).

Após análise comparativa dos resultados dos procedimentos psicométricos para os cinco modelos, decidiu-se cessar a eliminação de itens e apresentar a versão do KCL com dez itens como a versão mais adequada. Essa decisão foi baseada nas altas cargas fatoriais dos itens, poucos itens com baixos valores comuns, melhor ajuste por meio de índices de qualidade de ajuste e bons índices de confiabilidade. Além disso, o modelo bidimensional com dez itens preservou melhor sua estrutura de conteúdo, mantendo a coerência conceitual com o constructo de fragilidade no contexto oncológico. A Tabela 3 apresenta a análise fatorial dos modelos 4 e 5.

**Tabela 3:** Análise comparativa dos modelos 4 e 5 após análise fatorial exploratória, São Paulo, São Paulo, Brasil

Item	Modelo 4				Modelo 5		
	Carga fatorial			Comunalidade	Carga fatorial		Comunalidade
	Fator 1	Fator 2	Fator 3		Fator 1	Fator 2	
1	0,84	0,07	-0,13	0,70	0,75	0,15	0,70
**2**	**0,94**	-0,06	-0,08	0,76	**0,88**	0,01	0,79
**3**	**0,68**	-0,17	0,22	0,53	**0,75**	-0,06	0,52
**4**	**0,60**	-0,22	0,16	0,36	**0,58**	-0,09	0,29
**5**	**0,43**	-0,13	0,16	0,21	-	-	-
**6**	**0,39**	**0,51**	-0,12	0,58	-	-	-
**7**	**0,49**	0,23	-0,03	0,41	-	-	-
**8**	**0,67**	0,21	-0,13	0,59	-	-	-
**9**	-	-	-	-	-	-	-
**10**	0,08	0,24	0,29	0,23	-	-	-
**11**	-	-	-	-	-	-	-
**12**	-	-	-	-	-	-	-
**13**	0,11	0,09	**0,39**	0,24	-	-	-
**14**	**0,52**	0,20	0,03	0,46	-	-	-
**15**	-	-	-	-	-	-	-
**16**	0,08	-0,13	**0,88**	0,81	-	-	-
**17**	**0,45**	0,02	0,25	0,38	**0,51**	0,17	0,38
**18**	-0,18	0,19	**0,64**	0,40	-	-	-
**19**	-0,16	**0,54**	0,07	0,24	-0,11	**0,64**	0,34
**20**	0,02	**0,49**	0,24	0,38	0,07	**0,57**	0,38
**21**	0,11	**0,84**	-0,18	0,77	-0,01	**0,81**	0,65
**22**	-0,01	**0,69**	0,11	0,52	-0,08	**0,80**	0,59
**23**	0,02	**0,62**	0,12	0,45	0,06	**0,57**	0,37

*Nota: todas as cargas fatoriais foram apresentadas. Estão destacadas em negrito as cargas ≥ 0,35; “ - ” indica que o item não foi incluído na análise fatorial do modelo apresentado.*

A análise paralela do modelo final indicou duas dimensões. A primeira foi denominada como “atividades instrumentais”, composta pelos itens 1, 2, 3, 4 e 17, e a segunda, “humor deprimido”, composta pelos itens 19, 20, 21, 22 e 23.

Assim, após análise das evidências da estrutura interna, surgiu uma nova versão do KCL para mensuração da fragilidade em pacientes oncológicos com duas dimensões, composta por dez itens (os primeiros cinco mensuram a dimensão “atividades instrumentais”, e os cinco últimos, a dimensão “humor deprimido”), considerada a melhor versão, que foi denominada *Kihon Checklist*-ONCO10 (KCL-ONCO10), apresentada no Quadro 1.

**Quadro 1:** Versão de dez itens do *Kihon Checklist* (KCL-ONCO10) para medir fragilidade em pacientes com câncer hospitalizados, São Paulo, São Paulo, Brasil

*Kihon Checklist* (KCL-ONCO10)	
**1**	Você consegue usar ônibus, trem ou outro meio de transporte sem necessidade de ajuda?
**2**	Você faz compras para o seu dia a dia sem necessidade de ajuda?
**3**	Você administra sua conta bancária sozinho (a)?
**4**	Você visita a casa de seus amigos?
**5**	Você faz ligações telefônicas sozinho, checando você mesmo o número de telefone?
**6**	Nas últimas 2 semanas, você tem se sentido insatisfeito com sua vida diária?
**7**	Nas últimas 2 semanas, você tem achado sem graça as atividades com as quais você se divertia antes?
**8**	Nas últimas 2 semanas, você tem sentido dificuldade ao fazer coisas que antes achava fácil de fazer?
**9**	Nas últimas 2 semanas, você tem se sentido sem utilidade para os outros?
**10**	Nas últimas 2 semanas, você tem se sentido cansado sem motivo?

## DISCUSSÃO

Na oncologia, o desenvolvimento da doença e as toxicidades relacionadas ao tratamento desafiam os processos homeostáticos orgânicos. Na pessoa idosa, tais processos podem ser ainda mais custosos quando ponderamos sobre sua maior vulnerabilidade à doença em virtude dos próprios processos da senescência^(23)^.

Na prática clínica diária, é comum o uso de escalas que avaliam o *status* de desempenho dos pacientes com câncer, como a KPS e o *Eastern Cooperative Oncology Group*, que, no entanto, apresentam limitações quando aplicadas às pessoas idosas. Essas escalas sub-representam o *status* funcional desses pacientes e têm menor concordância entre observadores, enquanto instrumentos de avaliação de fragilidade podem avaliar a resiliência e a reserva de uma pessoa em longo prazo^(24)^.

A presença da fragilidade em pessoas idosas com câncer está associada a inúmeros resultados negativos em saúde como quedas, declínio funcional a longo prazo, maior taxa de complicações cirúrgicas e readmissão hospitalar, quando identificada na alta hospitalar após cirurgia oncológica^(25)^, além de se relacionar à diminuição da qualidade de vida e agravos à saúde mental nos idosos sobreviventes de câncer^(26,27)^.

Nesse contexto, a fragilidade se torna um indicador de tolerância ao tratamento e diretriz para um plano de cuidados individualizado^(4)^. Por isso, este estudo defende o uso de instrumento de avaliação de fragilidade com propriedades métricas testadas em um contexto oncológico para garantir resultados mais confiáveis e fidedignos na prática clínica.

A validade de conteúdo do KCL foi estabelecida por meio da análise CVR, um indicador robusto proveniente das avaliações de especialistas sobre a adequação dos itens em termos de clareza, relevância teórica e pertinência prática. Segundo Lawshe, esse indicador garante a validade de conteúdo com confiança^(13,14)^.

Apesar de demonstrar uma solução fatorial adequada, revelou dimensões diferentes daquelas relatadas pelos autores do instrumento original, encontrando vários problemas estruturais. O modelo inicial identificou inúmeros itens com baixas cargas fatoriais e comunalidades. Algumas hipóteses para as baixas pontuações nos indicadores podem ser atribuídas a fatores intrínsecos à doença oncológica, que podem ser comuns e reduzem a variância das respostas. Isso destaca a importância de revisitar tais evidências, principalmente no que diz respeito à estrutura interna^(28)^.

Os achados revelaram que a estrutura ótima para o KCL compreende dez itens, distribuídos em duas dimensões (instrumental e psicológica), avaliando 66,48% da fragilidade em pacientes idosos com câncer, com fortes evidências de validade e confiabilidade. Para que essa versão seja devidamente utilizada, é recomendada testagem para associações com outras variáveis e consequência do teste, completando o arco de evidências de validade^(4,28)^.

### Limitações do estudo

Embora tenhamos encontrado uma solução fatorial favorável para o KCL-ONCO10, a instabilidade na replicabilidade do construto representa uma limitação. Recomenda-se, portanto, a replicação do estudo em amostras maiores e mais diversas para confirmar a estabilidade do modelo. Apesar disso, o KCL-ONCO10 demonstra boas evidências de validade e confiabilidade para avaliar pessoas idosas com perfis semelhantes.

### Contribuições para a área da enfermagem, saúde ou políticas públicas

Este estudo oferece um instrumento com boas evidências de validade, que contribui para a avaliação da fragilidade em pessoas idosas hospitalizadas com câncer, subsidiando o planejamento do cuidado pela equipe de enfermagem e fortalecendo decisões clínicas individualizadas. Além disso, o estudo avança nas boas práticas de validação de instrumentos, por utilizar técnicas psicométricas contemporâneas que reforçam a robustez dos resultados.

## CONCLUSÕES

A versão brasileira do KCL com 25 itens não apresenta boas evidências de validade de conteúdo e estrutura interna em pacientes idosos com câncer. No entanto, o KCL-ONCO10, compreendendo dez itens em duas dimensões (atividades instrumentais e humor deprimido), demonstra boas evidências de validade de conteúdo, estrutura interna e confiabilidade para avaliar fragilidade em pacientes idosos com câncer hospitalizados.

## Data Availability

Os dados de pesquisa estão disponíveis em repositório: https://doi.org/10.48331/SCIELODATA.YYI2XL.
